# The prevalence of eating difficulties in children and young people in England: A large, cross‐sectional school survey

**DOI:** 10.1002/jcv2.70111

**Published:** 2026-03-17

**Authors:** Clara Faria, The OxWell Study Team, Mina Fazel, Emma Soneson, Tamsin J. Ford, Simon White

**Affiliations:** ^1^ Department of Psychiatry University of Cambridge Cambridge UK; ^2^ Department of Psychiatry University of Oxford Oxford UK

**Keywords:** DAWBA, eating difficulties, eating disorder, epidemiology, mental health, prevalence, psychiatry

## Abstract

**Background:**

Accurate population prevalence estimates of eating difficulties in children and young people provide essential information for the design and implementation of prevention efforts. We aimed to (I) explore the proportion of students reporting eating difficulties in a large English secondary school sample, (II) analyse factors associated with increased odds of eating difficulties and (III) estimate a weighted prevalence of eating difficulties in England.

**Methods:**

19,797 students in school years 7–11 (aged 11–16 years) and 3037 in school years 12–13 (aged 16–19 years) from the OxWell Student Survey completed questions from the Development and Well‐Being Assessment. A further 2664 had answers imputed using multiple imputation by chained equations, resulting in *n* = 25,498 students. The survey happened during February and March 2023. Logistic regression models estimated associations between gender, year group, ethnic group and eating difficulties. For students in Years 7–11, we estimated the prevalence of eating difficulties weighted to England's population.

**Results:**

The most endorsed difficulty was students thinking they were fat when others said they were very thin (47.3%; 11,277/23,837) and the least endorsed was self‐induced vomiting (17.7%; 4203/23,748). Girls (aOR 3.1, 95% CI 2.9, 3.2) and gender diverse/gender non‐disclosing young people (aOR 3.3, 95% CI 2.9, 3.9) had higher odds of having eating difficulties compared to boys, with increasing odds in older year groups. The weighted prevalence of eating difficulties in students in school years 7–11 was 62.5% (95% CI: 61.8, 63.3).

**Conclusion:**

The findings show that eating difficulties are common in secondary school students with more than half of our sample self‐reporting at least one type of eating difficulty. These data provide insight for clinical services, and the high prevalence further suggests that early intervention in community settings may have a valuable role in reducing the demand on eating disorder services.

## Key points

**What is known?**

- Eating difficulties in children and young people (CYP) are a common phenomenon with an estimated global prevalence of 22.4% in a recent meta‐analysis. Accurate population prevalence estimates of eating difficulties in CYP could lead to better service planning and timely clinical input for CYP with Eating Disorders (EDs) at early stages.

**What is new?**

- Using recent (2023) data from a large and diverse school‐based sample, we demonstrate that eating difficulties are common in secondary school students with more than half of our sample self‐reporting at least one type of eating difficulty. Girls and gender‐diverse/gender non‐disclosing and older young people are particularly at risk.

**What is relevant?**

- Further research should focus on understanding the transition between eating difficulties to a clinically impairing ED, preferably in large, general population samples using validated and robust measures.
- Community‐based prevention and early intervention programmes for eating difficulties should be explored. Schools in particular may have an untapped potential for interventions in eating difficulties, as CYP will generally have at least one of their meals in that environment.

## INTRODUCTION

Eating disorders (EDs) have the highest associated mortality of all mental health conditions, account for considerable morbidity and are a current major public health concern among children and adolescents (Treasure et al., 2020). Prevalence estimates in general population samples are instrumental to service planning, but point prevalence estimates for EDs are heterogeneous. Estimates range from 0.89% in a nationally representative Iranian Survey (Mohammadi et al., 2020) to 22.2% in an Australian study (Mitchison et al., 2020). There was a global surge in clinical presentations of EDs during and in the aftermath of the COVID‐19 pandemic. In England, emergency and urgent referrals doubled, with a lesser but still substantial increase in routine referrals (Nicholls et al., 2023). In Australia, the number of children with Anorexia nervosa requiring hospital admission for nutritional rehabilitation increased by 104% whereas in the United States ED diagnoses in electronic records became more common throughout the COVID‐19 pandemic (Madigan et al., 2024). In the UK, a cohort study found a 514% increase in admissions related to EDs over the past decade (Ward et al., 2025).

The aetiology of EDs in CYP is complex and involves the interplay of many genetic, behavioural and environmental factors (Culbert et al., 2015; Treasure et al., 2020), but includes eating difficulties (Constantini et al., 2026; Jacobi et al., 2004; Yamamiya & Stice, 2023). Eating difficulties comprise dysfunctional eating behaviours and beliefs that lead to an increased likelihood of broader difficulties with eating. They may be a precursor for later disorder but are not in themselves sufficiently clinically impairing to meet diagnostic criteria for an ED (López‐Gil et al., 2023; Newlove‐Delgado et al., 2023). More specifically, feeling a pressure to be thin, body dissatisfaction and abnormal eating behaviour such as self‐induced vomiting or restriction predicted future Bulimia Nervosa and Binge ED onset in a longitudinal study of adolescent girls in the United States (Yamamiya & Stice, 2023). However, the concept of eating difficulties is not very well established in the literature, contributing to some researchers using the term interchangeably with EDs (Pennesi & Wade, 2016). Eating difficulties in CYP are a common phenomenon with an estimated global prevalence of 22.4% in a recent meta‐analysis (López‐Gil et al., 2023). Nevertheless, it is still important to monitor eating difficulties in CYP, not only because they can cause distress in themselves, but also because they have potential to evolve into EDs, particularly certain types such as self‐induced vomiting (Constantini et al., 2026; Yamamiya & Stice, 2023).

Moreover, there is an argument to be made regarding the measurement of eating difficulties, which could lead to earlier detection and intervention for CYP with EDs. Some researchers argue that similarly to other areas of mental health, the ED field should adopt an early intervention model of mental illness (Phillippou et al., 2025). Under this framework, early identification and treatment before frank symptoms or illness have developed are key components (McGorry & Mei, 2018).

In England, the most recent prevalence data of eating difficulties in CYP comes from the English Mental Health of Children and Young People Surveys (MHCYP), which followed CYP recruited through a complex probability sample designed to reflect the English population. The 2023 survey estimated that in 2023, 12.3% of 11–16‐year‐olds and 59.4% of 17‐ to 19‐year‐olds in England were living with eating difficulties compared to 6.7% and 44.6% in 2017, respectively (Newlove‐Delgado et al., 2023; Sadler et al., 2018). Eating difficulties are known to vary by demographic characteristics. For example, the prevalence of eating difficulties varies greatly with gender, with many studies reporting much higher numbers of girls and young women with eating difficulties compared to boys and young men (Newlove‐Delgado et al., 2023). Limited evidence suggests that transgender young people are also at higher risk (Diemer et al., 2015), although this is an emerging area of research and many studies do not assess gender identities outside of the binary.

This study examined findings from over 25,000 CYP, aged 11–19 years of age, who participated in the 2023 OxWell Student Survey to (I) explore the proportions of students reporting eating difficulties in a large, secondary school sample, (II) analyse factors associated with increased odds of eating difficulties and (III) estimate a weighted, national point prevalence of eating difficulties in secondary school students in England.

## METHODS

This study uses data from the 2023 wave of the OxWell Student Survey (‘OxWell’). OxWell is a repeated cross‐sectional online study, which recruits CYP typically aged 9–18 years in English primary schools, secondary schools, and further education (FE) colleges (Mansfield et al., 2021). Schools are invited through their local authority and are informed about the study via a webinar and an information pack. Participating schools inform parents and carers, who can opt their children out of the study if their child is younger than 16. The use of a parental opt‐out model increases the likelihood of participation by more vulnerable and traditionally underrepresented groups (Chartier et al., 2008) and encourages more honest responses (Mansfield et al., 2021). Students < 16 years of age provide assent to participate, and students ≥ 16 years of age provide their own informed consent without parental involvement. Survey length varies from 15 to 40 min depending on the survey version and on students' responses to gateway questions. The current analysis used data from students in school years 7–13 (ages 11–19) who completed the 2023 survey.

The 2023 OxWell survey was granted ethical approval by the University of Oxford Research Ethics Committee (Reference: R62366/RE014).

### Measures

#### Eating difficulties

**FIGURE 1:**
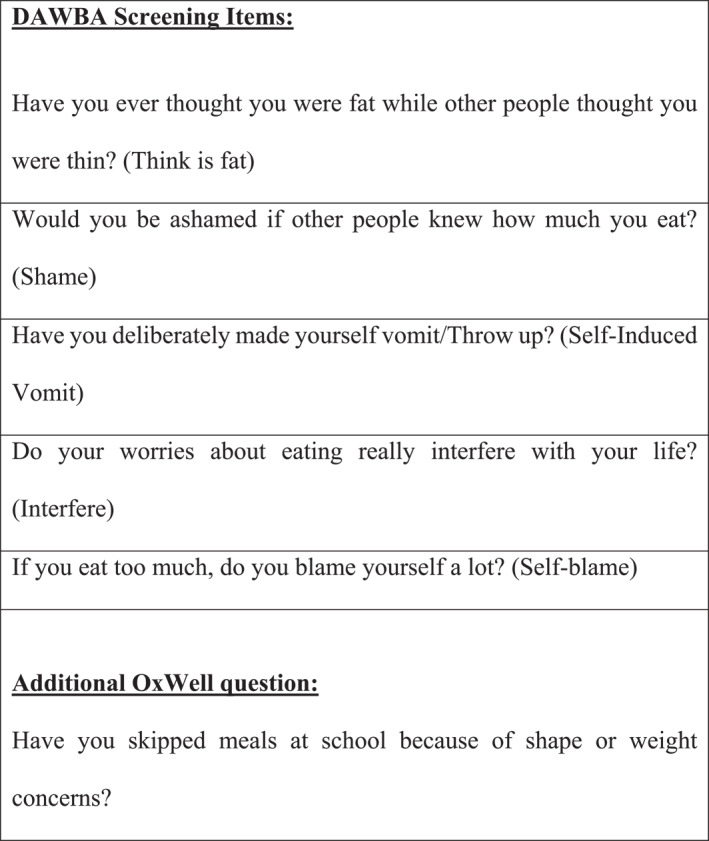
Eating difficulties questions from the DAWBA eating disorders module included in the 2023 OxWell Student Survey. DAWBA, Development and Well‐Being Assessment; EDs, eating disorders.

As a measure of eating difficulties, OxWell contains the five screening questions from the ED module of the Development and Well‐Being Assessment (DAWBA; Goodman et al., 2000), a multi‐informant standardised diagnostic measure (Figure 1), alongside an additional bespoke item about skipping meals at school because of shape or weight concerns. Each item had binary presence/absence (‘yes’/‘no’) responses. Due to the non‐identifiability of the survey design and the focus on a broad range of public mental health topics within a limited‐length questionnaire, the structured diagnostic follow‐on questions from the DAWBA were not included; hence only the DAWBA ED screening items were available to assess eating difficulties. Outcomes were based solely on the DAWBA screening items (i.e., exclusive of the bespoke item). The bespoke item on skipping meals was not included in the outcome as it had not been previously validated.

#### Demographic variables

Demographic variables included age, year group, gender, ethnicity, and socioeconomic deprivation of the school area. Age was measured in one‐year increments from 11 to 19. Year group ranged from Year 7 (11–12 years) to 13 (typically 17–18 years). Gender was coded as “Boy”, “Girl”, and “Gender Diverse/Gender Non‐Disclosing” (GD/GND) in accordance with the procedures outlined in Soneson et al. (2024). Ethnicity was collapsed into five groups (Asian, Black, Mixed or Multiple, White or Other), according to ONS Census Classification 6a (Office for National Statistics, 2022). Socioeconomic deprivation of the school postcode was assessed through the Index of Multiple Deprivation (IMD), which was derived from the UK Government IMD Database and is based on the Lower‐layer Super Output Area, using quintiles from 1 (most deprived) to 5 (least deprived).

## DATA ANALYSIS

**FIGURE 2:**
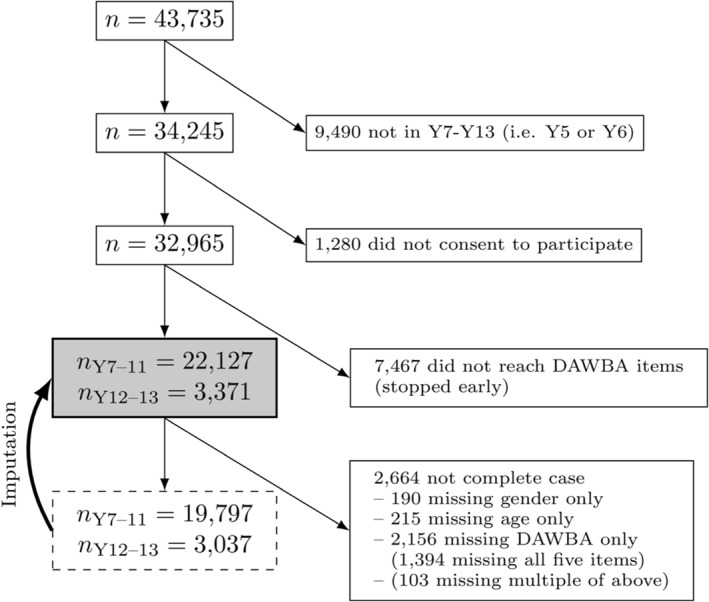
OxWell Student Survey 2023 sample flowchart. The dashed box indicates the complete‐case sample. The shaded box indicates the main analyses sample, using imputation to include 2664 additional CYP. CYP, children and young people.

The OxWell survey was started by 43,735 students, of whom 34,245 were in years 7–13 (Figure 2). Of these, 1280 participants were excluded because they did not provide consent to participate. Out of the remaining 32,965, 7467 stopped answering the survey before reaching the page containing the eating difficulties questions, whilst 19,797 students from year 7–11 and 3371 from year 12–13 completed all the DAWBA and demographic questions (the ‘complete‐case sample’). Those who reached the appropriate page with incomplete responses to the eating difficulties questions or missing demographics had answers imputed, resulting in our final sample size of 22,127 year 7–11 and 3371 year 12‐13 students (see Supporting Information S1: Appendix S4 for a sensitivity analysis including those who left the survey before the relevant items). We used multiple imputation by chained equations (MICE; Azur et al., 2011) to impute missing data on gender, age, the eating difficulties items and additional meal skipping item, which enabled us to calculated weighted prevalence using information from the publicly available Department for Education (DfE) school census data (Schools, Students and Their Characteristics, Academic Year 2023/24, [s.d.]). A complete case analysis may result in unrepresentative samples, and consequent selection bias, as well as a greatly reduced sample size (i.e., greater uncertainty). We decided to impute gender and age, because the OxWell Student Survey's weights are based on gender, age, and IMD population strata from the United Kingdom's DfE School Census. We did not impute ethnicity due to high missingness in this variable and because the information available in the dataset was insufficient to robustly impute ethnicity. For a detailed explanation of the imputation model, see Supporting Information S1: Appendix S1.

First, we describe the frequency of eating difficulties by demographic characteristics and the bespoke item on skipping meals at school and explore patterns of endorsement in the complete case dataset. Second, for the imputed dataset, we used multivariable logistic regressions to assess the associations with eating difficulties using a binary outcome indicating whether one or more items of the DAWBA items were endorsed. The bespoke item on skipping meals was not included in the outcome as it had not been previously validated. We ran separate models for students in Year 7–Y11, those in Year 12–13, and an additional model with all participants. We elected to analyse and report findings separately for these age groups to facilitate comparisons and synthesis with estimates from the MHCYP data (Newlove‐Delgado et al., 2023), which provide some of the most rigorous national data on eating difficulties amongst CYP.

Third, we calculated the weighted prevalence of eating difficulties among CYP in school‐based education, again explored by demographic characteristics. The weighted prevalence estimates are limited to the 11–16‐year‐old age group (year groups Y7–Y11). The OxWell weights are derived from the UK DfE School Census (Schools, Students and Their Characteristics, Academic Year 2023/24, [s.d.]); coverage of education establishments for the 17–19‐year‐old age group in the census does not include standalone FE colleges, whereas OxWell does include FE colleges; this mismatch between the study sampling frame and population reference makes weighting infeasible. Regarding mapping gender to the DfE School Census, we mapped the OxWell proportion across the school totals within each stratum. That is, we obtained Age‐IMD strata, then applied a 47.6/48/4.4 Male/Female/GD + GND split within each stratum to obtain the Gender‐Age‐IMD population counts (which are used to obtain the weights). Therefore, we are making Age‐IMD aligned prevalence estimates. For a full explanation of how survey weights were calculated, see Supporting Information S1: Appendix S2. We also conducted a sensitivity analysis to incorporate missing participants (see Supporting Information S1: Figure S1, Appendix S3 and S4).

## RESULTS

Overall, 63.1% reported at least one type of eating difficulty according to the DAWBA items. Table 1 illustrates the proportions of the OxWell sample endorsing each question about eating difficulties; 17.7% of the sample reported making themselves deliberately vomit, which was the least endorsed difficulty for both age groups (See Supporting Information S1: Table S1 for version including numerators and denominators). The most common difficulty reported was thinking one was fat even when others told them they were thin; 47.3% reported it. 32.6% of the OxWell sample said they would be ashamed if others knew how much they ate. Nearly all students (98.0%) who reported skipping meals also reported at least one type of eating difficulty in the DAWBA items. All eating difficulties were more frequently endorsed by girls and GD/GND students than boys. For example, GD/GND students had more than double the frequency of self‐induced vomit compared to boys (29.7% for GD/GND students vs. 20.7% for girl's vs. 12.8% for boys).

**TABLE 1:** Descriptive summary of responses to eating difficulties questions (non‐imputed dataset, hence each percentage has a different denominator due to missingness; see Supporting Information S1: Table S1 for numerators and denominators).

Group	Covariate	Total	Think is fat	Shame	Self‐induced vomit	Interfere	Self‐blame	Any eating difficulties
Gender	Boy	11,212	30.4%	17.7%	12.8%	20.8%	25.9%	49.1%
	GD/GND	1143	56.7%	54.4%	29.7%	49.8%	54.4%	76.3%
	Girl	12,895	61.0%	43.6%	20.7%	46.6%	51.0%	74.2%
	No response	248	53.8%	38.6%	32.2%	41.1%	44.2%	67.8%
Gender and year group	Boy in Y07‐11	9707	30.3%	17.7%	12.7%	20.6%	26.0%	49.1%
	GD/GND in Y07‐11	983	57.8%	54.6%	29.5%	50.2%	54.5%	76.8%
	Girl in Y07‐11	11,220	59.9%	42.7%	19.8%	45.7%	49.8%	73.3%
	Boy in Y12‐13	1505	31.3%	17.4%	13.5%	22.2%	25.6%	49.4%
	GD/GND in Y12‐13	160	50.7%	53.5%	31.2%	47.2%	53.5%	73.4%
	Girl in Y12‐13	1675	68.2%	49.5%	26.7%	52.2%	58.5%	80.1%
	No response (gender)	248	53.8%	38.6%	32.2%	41.1%	44.2%	67.8%
Ethnicity	White	13,581	49.5%	34.7%	18.5%	36.5%	42.2%	64.6%
	Mixed/multiple ethnic groups	1387	50.3%	36.3%	17.4%	37.4%	42.5%	66.3%
	Asian/Asian British	3657	43.4%	28.4%	13.4%	33.2%	35.1%	60.4%
	Black/Black British/African/Caribbean	1179	40.6%	26.6%	16.5%	30.4%	32.7%	57.7%
	Other ethnic group	994	44.8%	28.9%	17.0%	32.9%	37.7%	61.6%
	No response	4700	45.0%	31.2%	19.2%	34.5%	39.0%	61.7%
Year	Y07	5305	45.1%	30.8%	14.4%	31.2%	36.5%	61.1%
	Y08	5055	45.8%	32.1%	15.8%	34.5%	39.1%	62.0%
	Y09	4850	47.6%	33.4%	18.5%	36.5%	40.5%	63.8%
	Y10	3525	46.7%	31.6%	17.5%	34.7%	40.3%	62.2%
	Y11	3392	49.6%	33.6%	21.4%	38.6%	42.3%	65.1%
	Y12	2060	51.2%	36.0%	21.3%	39.9%	44.7%	65.6%
	Y13	1311	50.2%	34.3%	20.7%	36.5%	41.9%	66.1%
Skip meals	Yes	5688	82.1%	71.1%	45.5%	80.1%	84.1%	98.0%
	No	17,973	36.0%	20.3%	8.9%	21.0%	25.8%	52.1%
	No response	1837	65.0%	48.6%	23.1%	57.2%	65.1%	79.5%
Overall		25,498	47.3%	32.6%	17.7%	35.3%	40.0%	63.1%

*Note*: Percentage endorsing each of the five Development and Well‐Being Assessment (DAWBA) eating disorder screening items and percentage of any eating difficulties, that is endorsing at least one item (note, this column includes only participants with complete DAWBA data); percentages are presented overall, by gender, by year group, by gender and year group, by ethnicity, and by the additional OxWell item on skipping meals.

**FIGURE 3:**
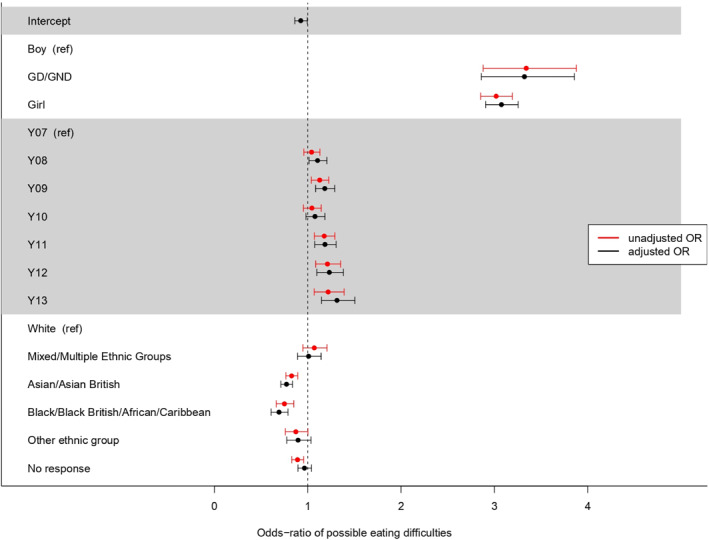
Odds‐ratios for any eating difficulties (endorse at least one of the DAWBA items, see Table 1) by gender, year group, and ethnicity in the imputed dataset. One adjusted logistic regression is fitted on all participants; the appropriate unadjusted model is also shown. The reference category for each covariate is indicated. Ethnicity was not imputed, hence the no response category (*n* = 4,700, see Table 1). Point estimate and 95% confidence interval are shown. (See Supporting Information S1: Table S2 for estimates). DAWBA, Development and Well‐Being Assessment.

Logistic regression models tested associations between demographic characteristics and eating difficulties for the whole sample and separately for 11–16‐year‐olds and for 17–19‐year‐olds. All models had unadjusted models fitted to explore the associations between gender, year group and ethnicity (see Figure 3 and Supporting Information S1: Figure S2).

In the unadjusted models, girls and GD/GND students had higher odds of having any eating difficulties compared to boys (OR 3.42 – CI 2.90, 4.01 for GD/GND students in Y7–11 and 2.94 – CI 1.99,4.29 ‐ for GD/GND students in Y12–13; OR 2.89 – CI 2.73, 3.07—for girls in Y7–11 and OR 4.07 – CI 3.46, 4.79 for girls in Y12–13; see Supporting Information S1: Figure S2 and Table S2). Compared with White students, those from Asian/Asian British and Black/Black British/African/Caribbean ethnic backgrounds, had lower odds of presenting with eating difficulties (OR 0.75 – CI 0.66, 0.86) for Black/Black British/African/Caribbean students in Y7–11 (OR 0.84 – CI 0.78, 0.92) and for Asian/Asian British students in Y7–11 0.72 95% CI 0.57, 0.90 for those in Y12–13). There was a small year group effect in the unadjusted model; students in Year 11 were more likely to present with eating difficulties compared to their reference group, Year 7 (OR 1.17 – CI 1.07, 1.29). The same small effect was not observed in the Y12–Y13 unadjusted model; students in Year 13 had an OR of 1.00 (CI 0.86, 1.16).

In the adjusted models, the pattern of associations remained broadly similar. Girls and GD/GND students in both age groups continued to show higher odds of eating difficulties compared to boys (OR 3.32 – CI 2.86, 3.85) for all GD/GND students, (OR 3.40 – CI 2.89, 3.99) for GD/GND students in Y7–11 and (OR 2.94 – CI 1.99, 4.31) for GD/GND students in Y12–13. For all girls, (OR 3.07 – CI 2.90, 3.25), for girls in Y7‐11 (OR 2.94 – CI 2.77, 3.12) and (OR 4.28 – CI 3.63, 5.05, for girls in Y12–13). Some ethnic differences were slightly more pronounced: students from Asian/Asian British backgrounds in Y7–Y11 and in Y12–13 had even lower adjusted ORs (0.80 and 0.56, respectively) relative to White students. For Black/Black British/African/Caribbean students, the association was only significant for those in Y7–Y11 (OR 0.70 – CI 0.61, 0.80). A year group gradient was also observed in the adjusted model; students in Year 11 (OR 1.19 – CI 1.08, 1.31) were still more likely to present with eating difficulties compared to their reference group, Year 7. The same association was observed for Year 9 (OR 1.18 – CI 1.08, 1.29) and Year 8 (OR 1.10 – CI 1.01, 1.20). Interestingly, there was no evidence of a difference for the older students– students in Year 13 had an OR of 1.08 (CI 0.92, 1.27), compared to their reference, students in year 12.

**FIGURE 4:**
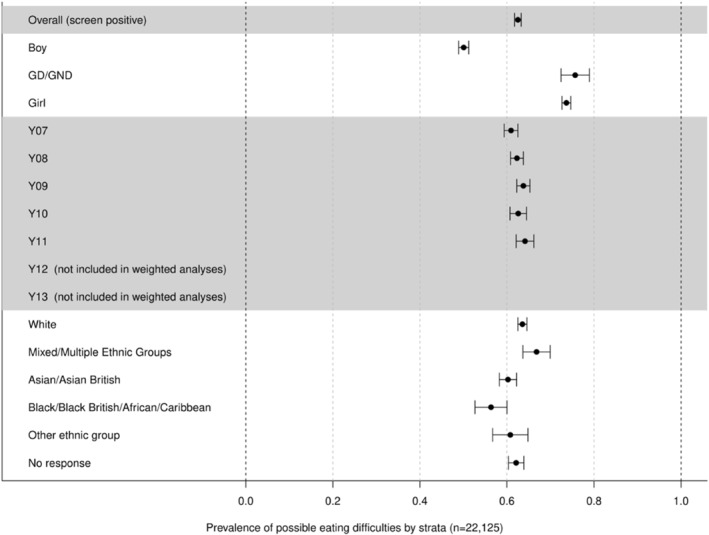
Survey‐weighted multiple imputation prevalence estimates of any eating difficulties among 11–16‐year‐olds (year groups 7–11). Prevalence is presented overall, by gender, by year group, and by ethnicity (all categories are independent of each other). Point prevalence and 95% confidence intervals are shown. (See Supporting Information S1: Table S3 for estimates).

The weighted point prevalence of eating difficulties for 11–16‐year‐olds was 62.5% (95% CI – 61.8, 63.3). The weighted point prevalence estimates for eating difficulties by subgroup are shown in Figure 4 and in Supporting Information S1: Table S3. Similarly to the raw proportions, girls and GD/GND students displayed increased prevalence of eating difficulties compared to boys. Regarding ethnic groups, students from White and Mixed/Multiple ethnicities had higher prevalences of eating difficulties. The prevalence increased slightly by year group (see Figure 3). For the sensitivity analysis results, see Supporting Information S1: Appendix S4 and Figures S1, S3–S8 and Tables S4 and S5.

## DISCUSSION

We analysed the proportion of students with eating difficulties and their associated demographic characteristics in the OxWell Student Survey and applied population weights to estimate national point prevalences, overall and by a number of subgroups. Eating difficulties were common among secondary school students, particularly girls and those who are gender diverse or who actively chose not to share their gender, with over two thirds of girls and GD/GND students reporting at least one type of eating difficulty. We found girls and GD/GND students to be at increased odds of displaying eating difficulties compared to boys. We also found that students in higher year groups were more likely to present with eating difficulties compared to their younger peers.

OxWell reported a higher prevalence of eating difficulties than the National Survey (MHCYP), which could relate to sociodemographic and methodological differences. OxWell reported 63.1% of students aged 11‐18 years‐old had at least one eating difficulty whilst MHCYP reported 12.3% of 11–16‐year‐olds and 59.4% of 17–19‐year‐olds reported the same. The MHCYP used solely the parent DAWBA report to estimate the prevalence of eating difficulties and EDs in children (aged 11–16), while in OxWell only the child report is utilised. The psychometric studies involving the DAWBA EDs screening module established a threshold of one positive answer out of five for a child or young person to be considered to have possible eating difficulties, whilst for the parental report, the threshold is two positive answers. The fact that the threshold for parents is higher and that in MHCYP, for some children aged 11–16 the only information available was the parent report, might have underestimated the level of difficulty experienced by their child. Also, parents might have been unaware of certain eating difficulties experienced by their child due to the ‘ego‐syntonic’ nature of some eating psychopathology (Treasure et al., 2020).

Previous literature has consistently highlighted that girls are more likely to report eating difficulties compared to boys. For instance, a systematic review and meta‐analysis conducted by López‐Gil et al. (2023) on the global proportions of disordered eating reported girls were more likely to present disordered eating than boys. In OxWell the gender groups at highest risk for disordered eating behaviours were GD/GND students and girls. OxWell provides a unique opportunity to report on GD/GND students, which has not, to our knowledge, previously been reported in a large‐scale survey. Our findings regarding their increased risk for eating difficulties underscore the importance of bringing attention to the unique needs and experiences of this group and echo broader calls to improve the measurement of gender diversity in youth surveys, in partnership with these young people (Soneson et al., 2024).

Another important consideration is what the high prevalence of eating difficulties is really capturing. In calculating whether a student had ‘any eating difficulties’, the five DAWBA screening items were weighted the same; however, the type of concerns and behaviours they capture are very different. For example, one of the questions is “Have you ever made yourself deliberately vomit or throw up”? which arguably reflects a higher level of disordered eating behaviour compared to the question “Have you ever thought you were fat while other people thought you were thin”? When comparing percentages, while 63.1% of the OxWell sample answered yes for at least one question, only 17.7% said yes to the vomiting question. It is also important to highlight that the DAWBA was originally designed for parental report as well, which we could not reproduce in this study, and this might have had an impact on the instrument's properties. The other caveat regarding the DAWBA's properties is there is increasing evidence that factors associated with the onset of EDs in ethnic minority groups may be distinct from those in white populations (Lewis et al., 2025). Researchers in disordered eating and ethnic minorities suggest that there might be more relevant factors, for example, conflict due to bicultural identity and lower motivation to eat in non‐White British groups, which arguably are not well captured by the DAWBA (Lewis et al., 2025). Therefore, our finding that eating difficulties were less likely in pupils from Asian/Asian British and Black/Black British/African/Caribbean ethnic backgrounds might not be capturing how these groups experience disordered eating.

Moreover, even though the DAWBA has been considered superior to other diagnostic interviews such as the EDE in clinical populations (House et al., 2008), we do not have studies comparing its properties to other diagnostic instruments in general populational samples. At a wider societal level, our findings indicate there is a shift in behaviour and body views among adolescents in 2023, which differs from findings reported by pre‐pandemic studies (Mishina et al., 2024). Moreover, our findings offer a snapshot of current adolescents' views on their relationship with their bodies. We need further follow‐up studies to better understand if this is a snapshot of a particular cohort or if the elevated rates of eating difficulties found represent an enduring trend.

## IMPLICATIONS FOR PRACTICE

Eating difficulties are not equivalent to clinically impairing EDs. However, our finding that over half of young people may be experiencing some level of eating difficulties could be one of the factors contributing to the reported increase in EDs presentations in clinical services in England (Taquet et al., 2021). We need to understand the trajectory between eating difficulties and EDs to be able to efficiently develop and implement future interventions.

Beyond the clinic, there is a paucity of research regarding eating difficulties and EDs in the school environment, particularly encompassing ethnic minority groups. From a public health perspective, schools play a pivotal role in CYP's mental health as most CYP will spend substantial amounts of time in their schools (Abdinasir et al., 2019). Schools may have an untapped potential for interventions as CYP will generally have at least one of their meals in that environment. Besides, there is moderate evidence that school based mental health interventions can be effective in improving mental health literacy and reducing mental health stigma (Ma et al., 2023), two important barriers when accessing help for EDs.

## LIMITATIONS

Our study has notable strengths, including the large and diverse community‐based sample, use of validated measures, and nuanced analytical approach (particularly as regards missing data). We also note several limitations. Firstly, in terms of measurement, the DAWBA ED screening items when used as a standalone self‐report measure yielded a national weighted prevalence of over 60% with eating difficulties—this magnitude may indicate a need for more comprehensive screening measures for eating difficulties. And, whilst EDs were *not* the focus of this analysis, it would also be important to have a better delineation of the specific contribution of individual screening items as indicators of an ED.

Secondly, in terms of the sample, OxWell is not designed to be a national probabilistic sample, and the analyses only include mainstream schools. This convenience sampling approach may have introduced selection bias. However, the survey does include over 200 schools from 6 different regions of England and due to its large sample size, it was possible to reduce uncertainty when calculating confidence intervals for the proportion of CYP affected by eating difficulties. Thirdly, OxWell weights use the Spring 2023/2024 Department of Education Census as an external data source, (Schools, Students and Their Characteristics, Academic Year 2023/24, [s.d.]), completion of which is not compulsory for independent schools and FE colleges. The latter account for approximately 5% of all CYP attending school in England, so does not provide complete coverage of the national student population (Schools, Students and Their Characteristics, Academic Year 2023/24). Moreover, the census does not capture gender diversity, so we created an approximation for this group in OxWell. It is also important to note that due to low numbers we collapsed the categories gender diverse and prefer not to say. A detailed account of how OxWell captured gender diversity has been published in a separate study (Soneson et al., 2024).

Thirdly, in terms of missing data, 7467 students who participated in OxWell did not reach the DAWBA question page in the survey and had to be excluded. It is not possible to determine whether they did not reach this page due to time constraints or whether they made an active decision to stop, for example, due to previous sensitive questions—particularly as concerns about privacy, confidentiality, and data use are common amongst OxWell participants (Soneson et al., 2025). A sensitivity analysis (see Supporting Information S1: Appendix S4) investigated three scenarios for the participants who stopped early. The uninformative scenario showed no divergence from the main analysis of the overall prevalence of eating difficulties (62.6% and 62.5%); while scenarios with substantial informative missingness showed moderate differences (65.2% and 60.1%) (Supporting Information S1: Table S5).

## CONCLUSION

Eating difficulties are common in mainstream secondary school students, with more than half of our large and diverse school‐based sample self‐reporting at least one type of eating difficulty. Eating difficulties were more common among girls and gender diverse/gender non‐disclosing youth, as well as in older ages, which is consistent with the previous body of literature on the topic. Further research should focus on understanding the transition between eating difficulties to a clinically impairing ED, preferably in large, general population samples using validated and robust measures. Service commissioners and policymakers should be aware of the likely ongoing pressure on Children and Adolescent Mental Health (CAMHS) and ED services, and community‐based prevention and early intervention programmes for eating difficulties—particularly in schools—should be explored.

## AUTHOR CONTRIBUTIONS:

**Clara Faria**: Conceptualization; formal analysis; investigation; methodology; project administration; software; validation; visualization; writing—original draft preparation; writing—review and editing. **The OxWell Study Team**: Project administration; resources; validation; visualization; writing—review and editing. **Mina Fazel**: Conceptualization; funding acquisition; resources; supervision; validation; visualization; writing—review and editing. **Emma Soneson**: Conceptualization; funding acquisition; resources; supervision; validation; visualization; writing—review and editing. **Tamsin J. Ford**: Conceptualization; funding acquisition; resources; supervision; validation; visualization; writing—review and editing. **Simon White**: Data curation; formal analysis; investigation; methodology; software; validation; visualization; writing—review and editing.

## CONFLICT OF INTEREST STATEMENT

T.F.’s research group receives payments from Place2Be, a third‐sector organisation who deliver mental health training and interventions to schools across the UK, for research methodology consultation. The remaining authors have declared that they have no competing or potential conflicts of interest.

## ETHICAL CONSIDERATIONS

The OxWell Student Survey (2023) has been approved by the University of Oxford Research Ethics Committee (Reference: R62366/RE0014). The year of approval was 2022. Participating schools in the OxWell Student Survey inform parents and carers, who can opt their children out of the study if their child is younger than 16. The use of a parental opt‐out model increases the likelihood of participation by more vulnerable and traditionally underrepresented groups (Chartier et al., 2008) and encourages more honest responses (Mansfield et al., 2021). Students <16 years of age provide assent to participate, and students ≥16 years of age provide their own informed consent without parental involvement.

## Supporting information

Supporting Information S1

## Data Availability

The data used in this study are not publicly available. The study protocol and variable guides are available through the Open Science Framework (https://osf.io/sekhr/). Fully deidentified extracts of the data can be provided to academic research collaborators upon reasonable request after a review process by the research team to ensure that uses of the data fall under the remit of the intended purposes set out in privacy information and to prevent duplication of analyses. The data is not publicly available because of ethical and information governance restrictions. The full list of questions as well as other details are available on a project‐specific OxWell Open Science Framework website (https://osf.io/sekhr/) along with the study protocol.
