# Supplementary material for: The prevalence of eating difficulties in children and young people in England: A large, cross‐sectional school survey
Source: JCPP Adv. 2026 Mar 17:e70111. Online ahead of print. doi: 10.1002/jcv2.70111 (PMC13339529; doi:10.1002/jcv2.70111)
Supplement: Supplementary file 1 — Supporting Information S1 [file JCV2-9999-e70111-s001.docx]

**The prevalence of eating difficulties in children and young people in England: a large, cross-sectional school survey**

**Supporting Information**

**Appendix S1. Imputation**

The main analysis dataset (see Figure 1) includes missingness among age, gender, ethnicity, the five DAWBA ED module items, and the additional skip meal item; from the survey we have year group (which is complete as it was a component of the consent process) and IMD (obtained by linking the school postcode with the ONS LOSA statistics). As discussed in the main paper, we elect to leave ethnicity with a no response category, given the variables available there is no justifiable imputation model for ethnicity at an individual-level. We note that there is also minimal information to impute gender beyond the gender—age relationship with eating difficulties, but gender is necessary for the weighted analyses that follow whereas ethnicity is not.

Imputation was performed using MICE, assuming a Missing At Random (MAR) missingness model where an outcome may be missing due to observed covariates. We used a dense prediction matrix, with all variables included as predictors. We generated 250 imputed datasets, each based on 10 iterations of the chained equations (to ensure stability/convergence of each imputed dataset). The DAWBA ED items and skip meals item used a logistic imputation model, while age and gender used a multinomial logistic imputation model. Imputations were performed using the GNU R mice package and took approximately two hours to run. Imputations were checked for convergence using visual inspection of iteration plots from mice.

**Appendix S2. OxWell Student Survey Weights**

The United Kingdom Department for Education (DfE) conducts a statutory School Census, all state funded schools are required to report pupil counts and additional school-level statistics (for example, proportion of pupils receiving free school meals); independent schools are required to report pupil counts but without some cross-tabulations (for example, by fulltime/parttime).

The School Census can be taken as the reference population for OxWell, whose sampling frame covers all schools within the selected Local Education Authorities (LEAs). However, there are several important limitations to the School Census we should consider weighting OxWell to be representative. However:

- The DfE census is only for England (not Scotland, Wales, or Northern Ireland). Hence we cannot claim UK-representative
- The DfE census does not include standalone Further Education (i.e. colleges). Hence care must be taken when talking about these groups

Given these limitations, we construct the reference population as Children and Young People (CYP) participating in school-based education for year groups Y7 to Y11 (ages 11 to 16). The DfE Census reports total head count of boys and girls by age across schools. There are two complications to address with the reference population:

- Age or year group: The OxWell Student Survey is designed to sample by year group within participating schools, whereas the DfE School Census reports head count by age (year group counts are available for some schools, but not compulsory). Hence, we impute missing age in the OxWell dataset to obtain a weight for each participant. These weights will be biased for 16 year olds due to the conflating of school-based sixth forms (year groups 12 and 13), which are included in the DfE School Census; specifically the reference population will include too many 16 year olds (as some will be in Y12). If we were assessing prevalence in Y11 and Y12 this would be a bigger issue.
- The UK Government mandated that the DfE School Census recorded each student’s binary sex (female/male) “which identifies the sex of a person as recognised in law” (see <https://explore-education-statistics.service.gov.uk/methodology/schools-pupils-and-their-characteristics>). Given the focus of self-identified gender within OxWell, it was felt that we could not (and should not) map OxWell responses on to a binary female/male. Weights are based on population strata defined by age and IMD, with gender aggregates proportions being applied from the OxWell responses; that is, the DfE sex is not used only total head count and this is apportioned among the OxWell gender categories: Boy, GD/GND, and Girl as 47.6%, 4.4%, and 48% respectively.

The reference population is used to generate gender—age—IMD strata totals, which are used to derive survey weights applied to the OxWell participants. The weighting analyses are performed using the GNU R survey package, calculating overall prevalence and stratified prevalence estimates.

**Appendix S3. Additional results**

**Table S1. Version of Table 1 including numerators and denominators. Note, this is on the non-imputed dataset and hence the denominator of each percentage is different; and there are no response rows for gender and the skip meals question.**

| **Group** | **Covariate** | **Total** | **Think is fat** | **Shame** | **Deliberate vomit** | **Interfere** | **Self-blame** | **Screen positive** |
| --- | --- | --- | --- | --- | --- | --- | --- | --- |
| Gender | Boy | 11,212 | 30.4% | 17.7% | 12.8% | 20.8% | 25.9% | 49.1% |
|  |  |  | 3,186/10,472 | 1,847/10,451 | 1,337/10,454 | 2,169/10,420 | 2,699/10,422 | 5,065/10,309 |
|  | GD/GND | 1,143 | 56.7% | 54.4% | 29.7% | 49.8% | 54.4% | 76.3% |
|  |  |  | 589/1,038 | 561/1,031 | 306/1,029 | 512/1,028 | 558/1,026 | 763/1,000 |
|  | Girl | 12,895 | 61.0% | 43.6% | 20.7% | 46.6% | 51.0% | 74.2% |
|  |  |  | 7,389/12,117 | 5,278/12,101 | 2,493/12,057 | 5,604/12,035 | 6,134/12,036 | 8,712/11,740 |
|  | No response | 248 | 53.8% | 38.6% | 32.2% | 41.1% | 44.2% | 67.8% |
|  |  |  | 113/210 | 81/210 | 67/208 | 85/207 | 92/208 | 139/205 |
| G/Y | Boy.Y0711 | 9,707 | 30.3% | 17.7% | 12.7% | 20.6% | 26.0% | 49.1% |
|  |  |  | 2,747/9,068 | 1,603/9,050 | 1,148/9,051 | 1,858/9,019 | 2,342/9,025 | 4,381/8,923 |
|  | GD/GND.Y0711 | 983 | 57.8% | 54.6% | 29.5% | 50.2% | 54.5% | 76.8% |
|  |  |  | 514/890 | 484/887 | 261/885 | 444/884 | 482/884 | 661/861 |
|  | Girl.Y0711 | 11,220 | 59.9% | 42.7% | 19.8% | 45.7% | 49.8% | 73.3% |
|  |  |  | 6,315/10,543 | 4,502/10,532 | 2,078/10,500 | 4,787/10,470 | 5,221/10,476 | 7,487/10,210 |
|  | Boy.Y1213 | 1,505 | 31.3% | 17.4% | 13.5% | 22.2% | 25.6% | 49.4% |
|  |  |  | 439/1,404 | 244/1,401 | 189/1,403 | 311/1,401 | 357/1,397 | 684/1,386 |
|  | GD/GND.Y1213 | 160 | 50.7% | 53.5% | 31.2% | 47.2% | 53.5% | 73.4% |
|  |  |  | 75/148 | 77/144 | 45/144 | 68/144 | 76/142 | 102/139 |
|  | Girl.Y1213 | 1,675 | 68.2% | 49.5% | 26.7% | 52.2% | 58.5% | 80.1% |
|  |  |  | 1,074/1,574 | 776/1,569 | 415/1,557 | 817/1,565 | 913/1,560 | 1,225/1,530 |
|  | No response (Gender) | 248 | 53.8% | 38.6% | 32.2% | 41.1% | 44.2% | 67.8% |
|  |  |  | 113/210 | 81/210 | 67/208 | 85/207 | 92/208 | 139/205 |
| Ethnicity | White | 13,581 | 49.5% | 34.7% | 18.5% | 36.5% | 42.2% | 64.6% |
|  |  |  | 6,364/12,845 | 4,440/12,810 | 2,371/12,801 | 4,662/12,757 | 5,392/12,776 | 8,107/12,554 |
|  | Mixed/Multiple Ethnic Groups | 1,387 | 50.3% | 36.3% | 17.4% | 37.4% | 42.5% | 66.3% |
|  |  |  | 656/1,303 | 472/1,302 | 226/1,298 | 485/1,297 | 550/1,294 | 841/1,269 |
|  | Asian/Asian British | 3,657 | 43.4% | 28.4% | 13.4% | 33.2% | 35.1% | 60.4% |
|  |  |  | 1,487/3,423 | 973/3,425 | 459/3,417 | 1,135/3,415 | 1,198/3,411 | 2,021/3,347 |
|  | Black/Black British/African/Caribbean | 1,179 | 40.6% | 26.6% | 16.5% | 30.4% | 32.7% | 57.7% |
|  |  |  | 440/1,084 | 290/1,092 | 179/1,085 | 329/1,084 | 353/1,078 | 608/1,054 |
|  | Other ethnic group | 994 | 44.8% | 28.9% | 17.0% | 32.9% | 37.7% | 61.6% |
|  |  |  | 410/916 | 264/913 | 154/908 | 299/909 | 343/911 | 547/888 |
|  | No Response | 4,700 | 45.0% | 31.2% | 19.2% | 34.5% | 39.0% | 61.7% |
|  |  |  | 1,920/4,266 | 1,328/4,251 | 814/4,239 | 1,460/4,228 | 1,647/4,222 | 2,555/4,142 |
| Year | Y07 | 5,305 | 45.1% | 30.8% | 14.4% | 31.2% | 36.5% | 61.1% |
|  |  |  | 2,202/4,879 | 1,495/4,858 | 698/4,862 | 1,510/4,833 | 1,768/4,844 | 2,901/4,745 |
|  | Y08 | 5,055 | 45.8% | 32.1% | 15.8% | 34.5% | 39.1% | 62.0% |
|  |  |  | 2,170/4,739 | 1,517/4,721 | 746/4,712 | 1,623/4,709 | 1,841/4,706 | 2,854/4,606 |
|  | Y09 | 4,850 | 47.6% | 33.4% | 18.5% | 36.5% | 40.5% | 63.8% |
|  |  |  | 2,163/4,544 | 1,519/4,550 | 838/4,539 | 1,650/4,525 | 1,835/4,528 | 2,835/4,441 |
|  | Y10 | 3,525 | 46.7% | 31.6% | 17.5% | 34.7% | 40.3% | 62.2% |
|  |  |  | 1,552/3,320 | 1,051/3,321 | 579/3,312 | 1,145/3,300 | 1,329/3,300 | 2,014/3,236 |
|  | Y11 | 3,392 | 49.6% | 33.6% | 21.4% | 38.6% | 42.3% | 65.1% |
|  |  |  | 1,587/3,200 | 1,074/3,200 | 682/3,190 | 1,231/3,185 | 1,347/3,186 | 2,047/3,143 |
|  | Y12 | 2,060 | 51.2% | 36.0% | 21.3% | 39.9% | 44.7% | 65.6% |
|  |  |  | 985/1,923 | 689/1,914 | 406/1,906 | 762/1,908 | 852/1,908 | 1,230/1,875 |
|  | Y13 | 1,311 | 50.2% | 34.3% | 20.7% | 36.5% | 41.9% | 66.1% |
|  |  |  | 618/1,232 | 422/1,229 | 254/1,227 | 449/1,230 | 511/1,220 | 798/1,208 |
| Skip Meals | Yes | 5,688 | 82.1% | 71.1% | 45.5% | 80.1% | 84.1% | 98.0% |
|  |  |  | 4,652/5,663 | 4,016/5,646 | 2,556/5,618 | 4,517/5,637 | 4,742/5,637 | 5,409/5,517 |
|  | No | 17,973 | 36.0% | 20.3% | 8.9% | 21.0% | 25.8% | 52.1% |
|  |  |  | 6,434/17,880 | 3,627/17,892 | 1,592/17,892 | 3,746/17,866 | 4,618/17,866 | 9,177/17,620 |
|  | No response | 1,837 | 65.0% | 48.6% | 23.1% | 57.2% | 65.1% | 79.5% |
|  |  |  | 191/294 | 124/255 | 55/238 | 107/187 | 123/189 | 93/117 |
| Overall |  | 25,498 | 47.3% | 32.6% | 17.7% | 35.3% | 40.0% | 63.1% |
|  |  |  | 11,277/23,837 | 7,767/23,793 | 4,203/23,748 | 8,370/23,690 | 9,483/23,692 | 14,679/23,254 |

**Table S2. Odds Ratios for all age-ranges (11-16 & 17-19) used to create Figure 3**

| **Covariate** | | **SE (adjusted)** | | **SE (unadjusted)** | | **FE (adjusted)** | | **FE (unadjusted)** | | **All (adjusted)** | |
| --- | --- | --- | --- | --- | --- | --- | --- | --- | --- | --- | --- |
|  |  | **Estimate** | **CI** | **Estimate** | **CI** | **Estimate** | **CI** | **Estimate** | **CI** | **Estimate** | CI |
| Ethnicity | No response | 0.982 | (0.907, 1.063) | 0.907 | (0.840, 0.979) | 0.860 | (0.694, 1.066) | 0.805 | (0.658, 0.986) | 0.965 | (0.896, 1.040) |
|  | Other ethnic group | 0.913 | (0.785, 1.062) | 0.895 | (0.773, 1.035) | 0.785 | (0.497, 1.240) | 0.759 | (0.494, 1.166) | 0.897 | (0.776, 1.036) |
|  | Black/Black British/African/Caribbean | 0.698 | (0.609, 0.801) | 0.756 | (0.663, 0.863) | 0.668 | (0.447, 0.999) | 0.749 | (0.514, 1.089) | 0.693 | (0.609, 0.790) |
|  | Asian/Asian British | 0.803 | (0.737, 0.875) | 0.847 | (0.780, 0.920) | 0.566 | (0.446, 0.719) | 0.722 | (0.578, 0.901) | 0.773 | (0.713, 0.838) |
|  | Mixed/Multiple Ethnic Groups | 1.046 | (0.915, 1.194) | 1.116 | (0.982, 1.269) | 0.799 | (0.557, 1.146) | 0.817 | (0.582, 1.147) | 1.010 | (0.892, 1.144) |
|  | White |  |  |  |  |  |  |  |  |  |  |
| Year | Y13 |  |  |  |  | 1.086 | (0.925, 1.275) | 1.006 | (0.866, 1.170) | 1.314 | (1.147, 1.506) |
|  | Y12 |  |  |  |  |  |  |  |  | 1.232 | (1.098, 1.382) |
|  | Y11 | 1.185 | (1.076, 1.305) | 1.177 | (1.072, 1.292) |  |  |  |  | 1.185 | (1.076, 1.306) |
|  | Y10 | 1.076 | (0.979, 1.183) | 1.046 | (0.955, 1.145) |  |  |  |  | 1.078 | (0.981, 1.185) |
|  | Y09 | 1.182 | (1.084, 1.288) | 1.128 | (1.038, 1.226) |  |  |  |  | 1.183 | (1.085, 1.290) |
|  | Y08 | 1.103 | (1.012, 1.202) | 1.042 | (0.959, 1.132) |  |  |  |  | 1.106 | (1.015, 1.205) |
|  | Y07 |  |  |  |  |  |  |  |  |  |  |
| Gender | Girl | 2.943 | (2.770, 3.126) | 2.899 | (2.730, 3.078) | 4.284 | (3.631, 5.055) | 4.073 | (3.463, 4.791) | 3.076 | (2.906, 3.255) |
|  | GD/GND | 3.399 | (2.891, 3.997) | 3.418 | (2.908, 4.017) | 2.936 | (1.999, 4.314) | 2.925 | (1.994, 4.290) | 3.322 | (2.861, 3.857) |
|  | Boy |  |  |  |  |  |  |  |  |  |  |
| (Intercept) | Intercept | 0.934 | (0.868, 1.005) |  |  | 1.076 | (0.938, 1.236) |  |  | 0.926 | (0.862, 0.995) |

**Table S3. Weighted prevalence estimates of eating difficulties used to create Figure 4.**

| **Covariate** |  | **Estimate** | **CI** |
| --- | --- | --- | --- |
| Ethnicity | No response | 0.6211 | (0.6034, 0.6388) |
|  | Other ethnic group | 0.6077 | (0.5672, 0.6483) |
|  | Black/Black British/African/Caribbean | 0.5635 | (0.5267, 0.6002) |
|  | Asian/Asian British | 0.6023 | (0.5824, 0.6222) |
|  | Mixed/Multiple Ethnic Groups | 0.6681 | (0.6368, 0.6994) |
|  | White | 0.6355 | (0.6252, 0.6459) |
| Year | Y13 |  |  |
|  | Y12 |  |  |
|  | Y11 | 0.6416 | (0.6213, 0.6618) |
|  | Y10 | 0.6260 | (0.6070, 0.6449) |
|  | Y09 | 0.6378 | (0.6227, 0.6529) |
|  | Y08 | 0.6230 | (0.6082, 0.6377) |
|  | Y07 | 0.6094 | (0.5936, 0.6251) |
| Gender | Girl | 0.7366 | (0.7265, 0.7466) |
|  | GD/GND | 0.7568 | (0.7242, 0.7894) |
|  | Boy | 0.5007 | (0.4892, 0.5122) |
| Status | Overall (screen positive) | 0.6252 | (0.6175, 0.6328) |

**Appendix S4. Sensitivity analysis**

In Figure 1 we note that there are a number of participants that stopped before reaching the page of the survey containing the DAWBA items. In fact, there is a hierarchy of missingness as follows:

- (Complete) all DAWBA items answered
- (Incomplete) at least one DAWBA item answered
- (None) participant reached the page (or later) but did not answer any DAWBA items
- (Stopped) participant stopped before reaching the page so did not answer any DAWBA items – and further did not see the DAWBA items

The main analysis performs a Missing at Random (MAR) type imputation on the “Incomplete” and “None” participants, assuming that we can determine the missing values based on the observed covariates. This is reasonable for “Incomplete” and adequate for “None”.

In this section, we perform a sensitivity analysis to incorporate the “Stopped” participants. We conduct three imputations: (A) assuming MAR and treating the “Stopped” the same as the “None”; and (B) and (C) conducting a Missing Not At Random (MNAR) imputation.

**Figure S1. Sensitivity flowchart (see Figure 1)**


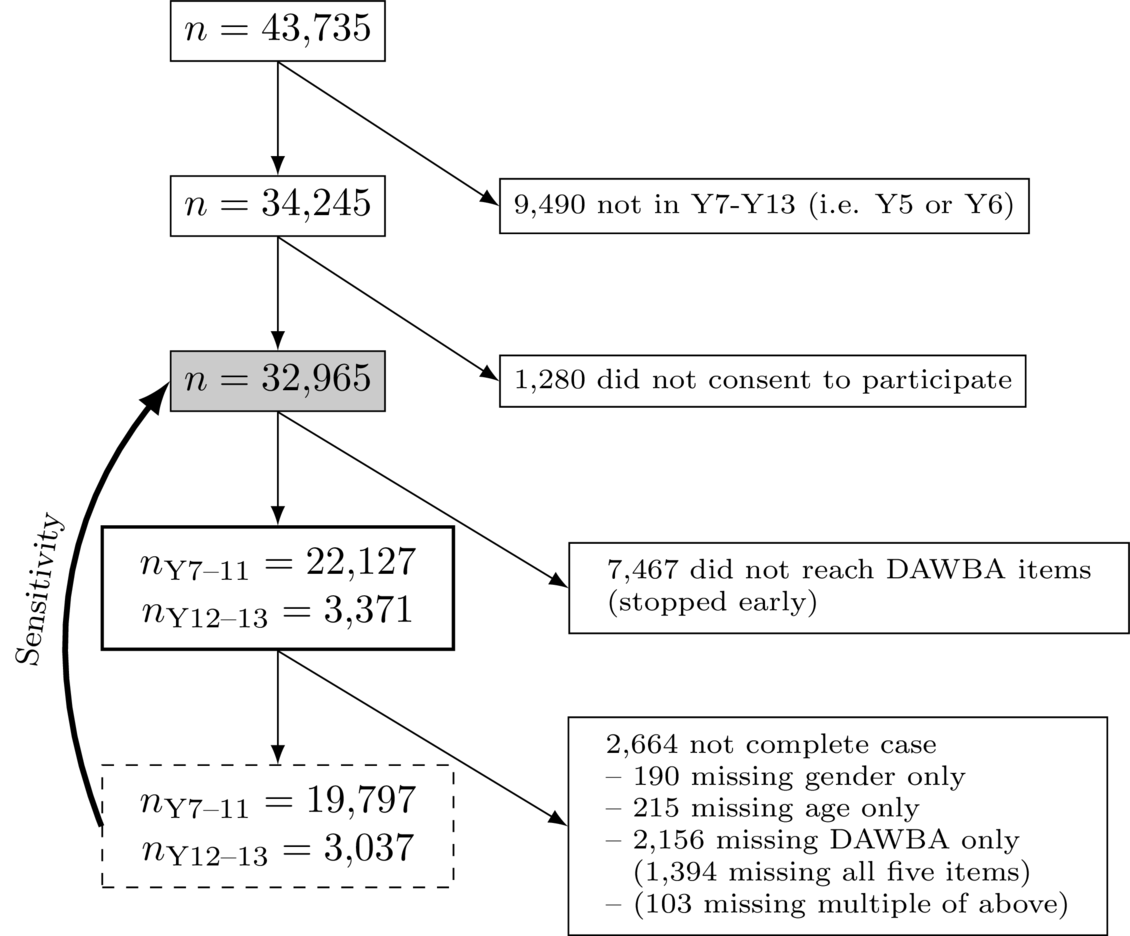


Missing Not at Random (MNAR) assumes that there is an effect outside the observed data that influences the outcome. Naturally, this cannot be learnt from the data itself and so a sensitivity analysis is conducted inducing an effect. We use a MNAR logistic regression for the DAWBA and skipped meal items, adding an auxiliary effect of stopping early (to differentiate “Stopped” from “None”). We consider two scenarios:

- (B) All missing DAWBA and Skip Meals items are 22% more likely (log-odds 0.2) to be “Yes” for those who “Stopped”. This reflects the view that people who stop early may have worse overall well-being.
- (C) All missing DAWBA and Skip Meals items are 19% less likely (log-odds -0.2) to be “Yes” for those who “Stopped”. This reflects the view that people who stop early may feel the OxWell Student Survey is not relevant to them.

Both scenarios are simplistic, recall that “Status” is positive if at least one DAWBA ED item is endorsed meaning that the above scenarios impact all five DAWBA items and compound the impact. The resulting equivalent results are presented below (scenario A in effect sets the MNAR logit effect to be zero on the log-odds scale).

**Figure S2. Odds-ratios for any eating difficulties (endorse at least one of the DAWBA items, see Table 1) by gender, year group, and ethnicity in the imputed dataset (n=22,127 and n=3,371 for Y7-11 and Y12-13 respectively). Three adjusted logistic regressions are fitted on Y7-11 only, Y12-13 only, and all participants; the appropriate unadjusted models are also shown (for visual clarity, the unadjusted model intercepts are omitted). The reference category for each covariate is indicated; note the varying year group reference across the models. Ethnicity was not imputed, hence the no response category (n=4,700, see Table 1). Point estimate and 95% confidence interval are shown. (See Table S2 for estimates).**

**
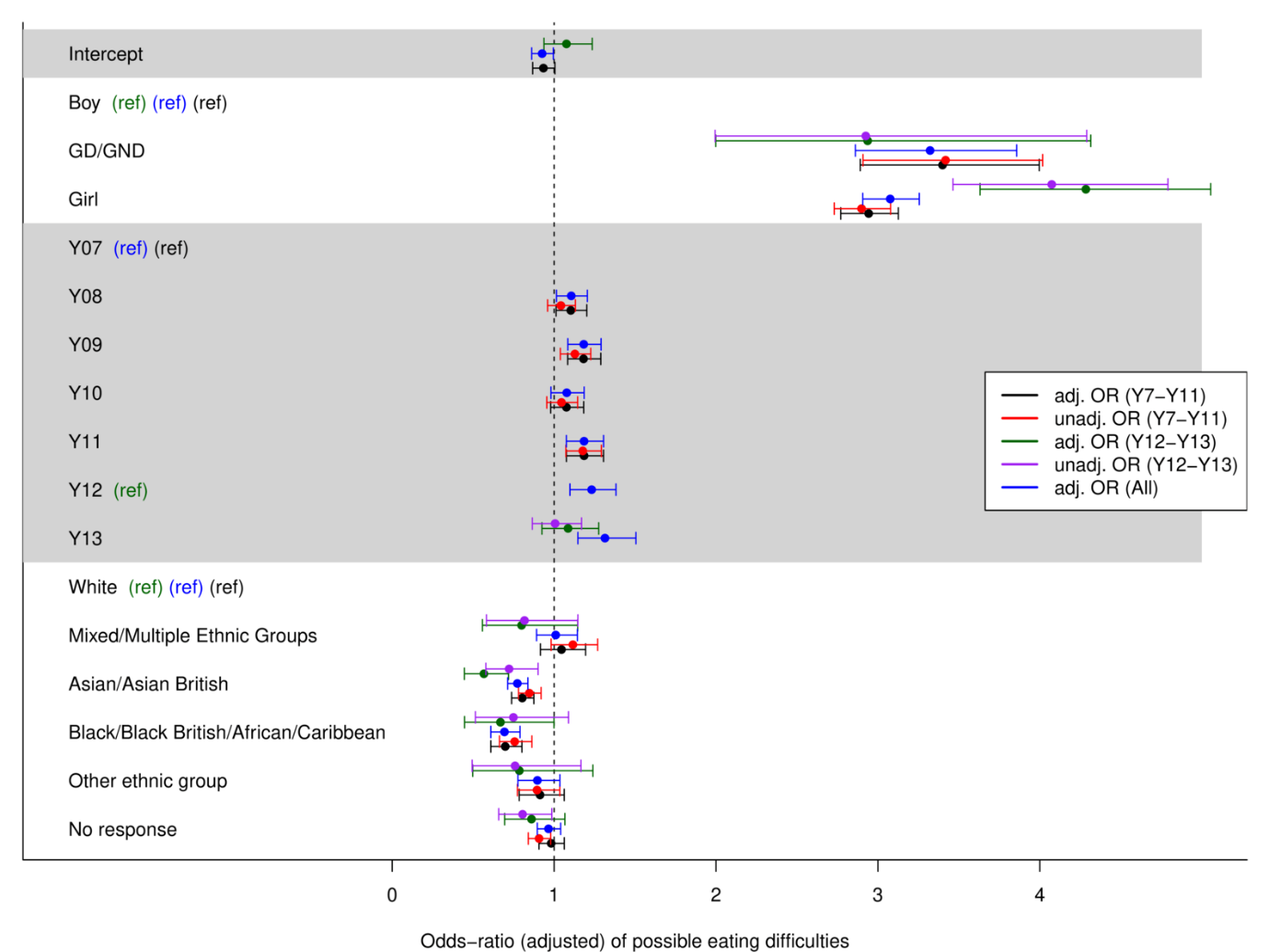
**

**Figure S3. Sensitivity Scenario B – all models**

**
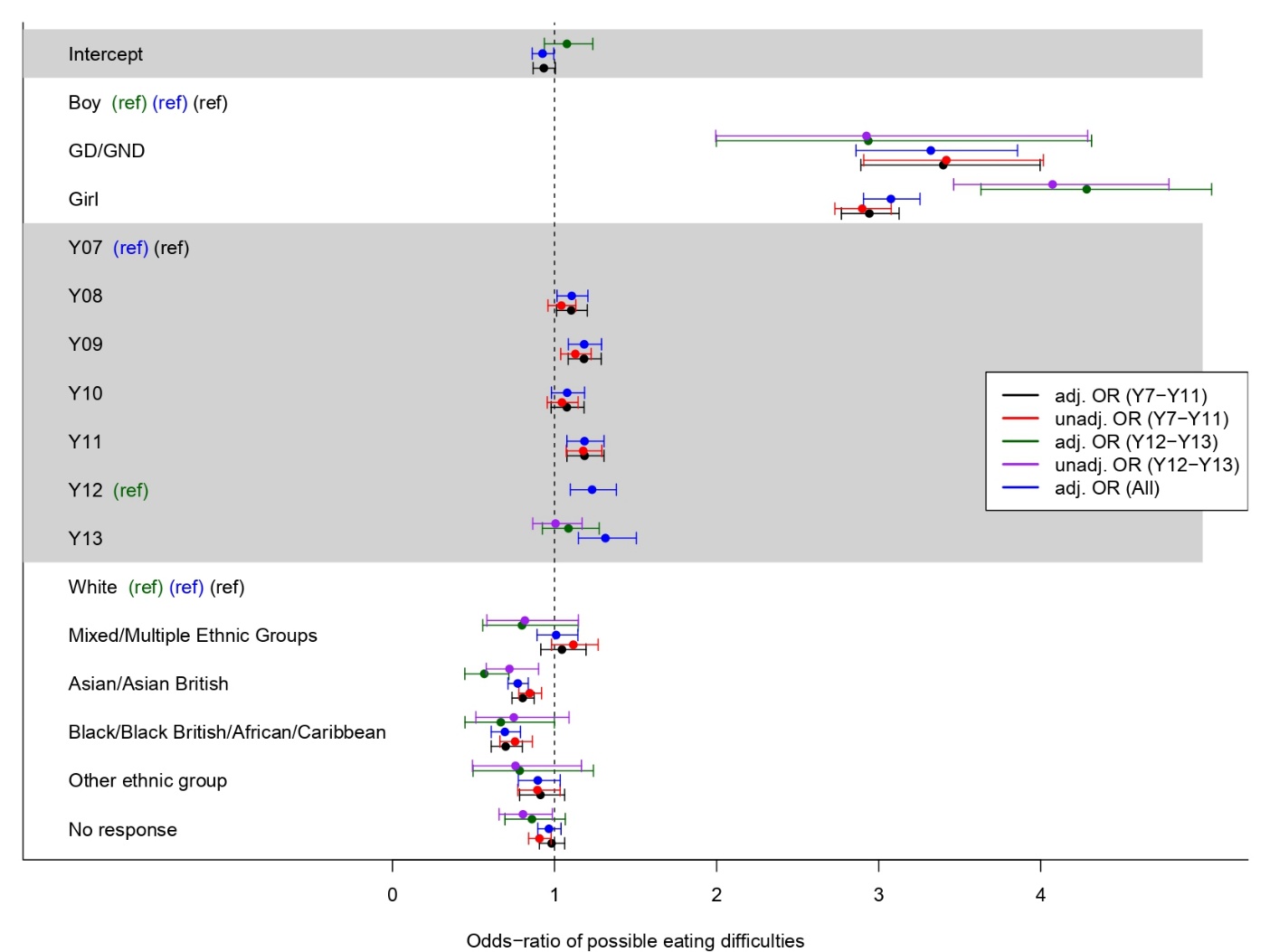
**

**Figure S4. Sensitivity Scenario A – all models**


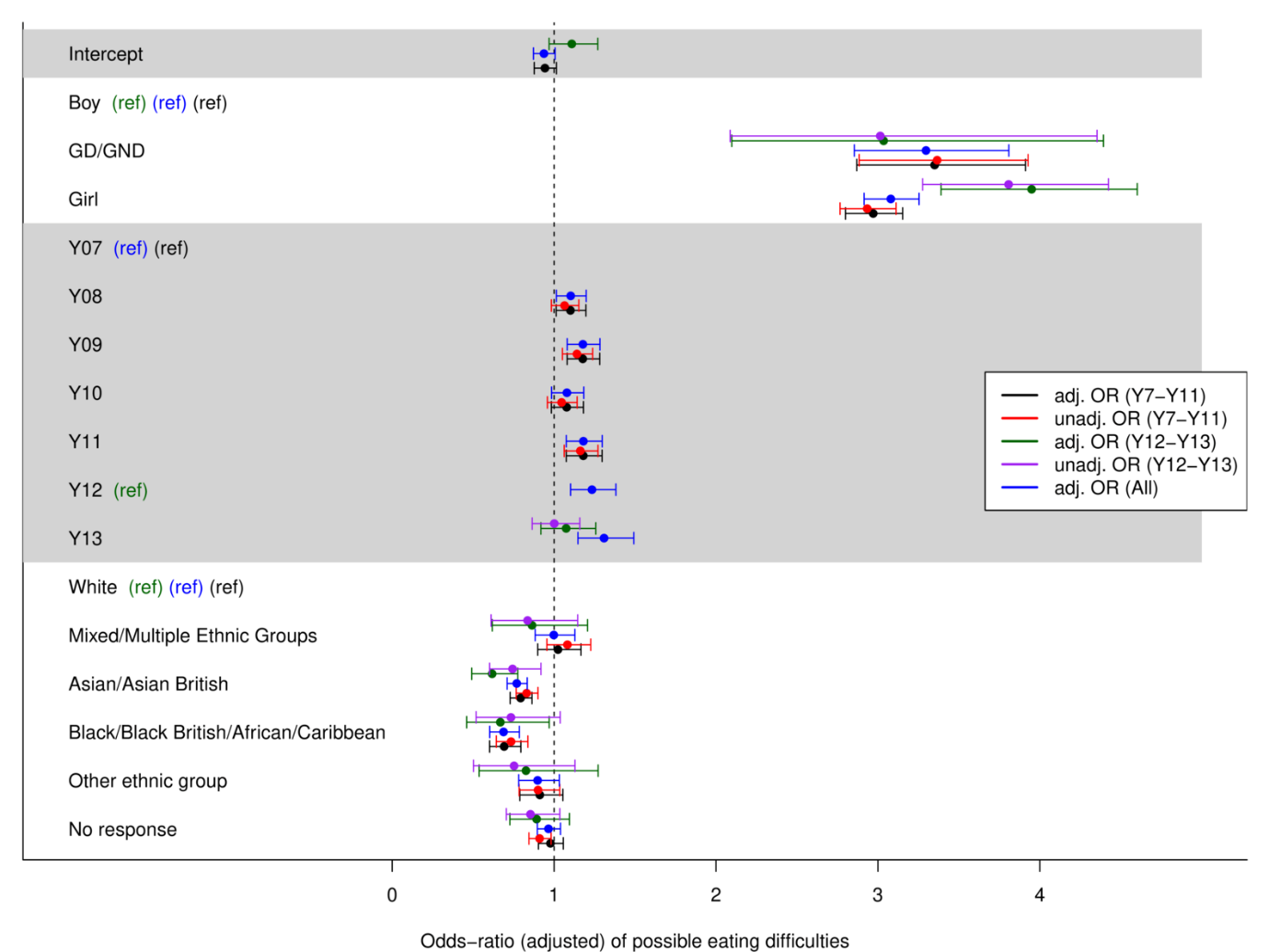


**Figure S5. Sensitivity Scenario C – all models**

**
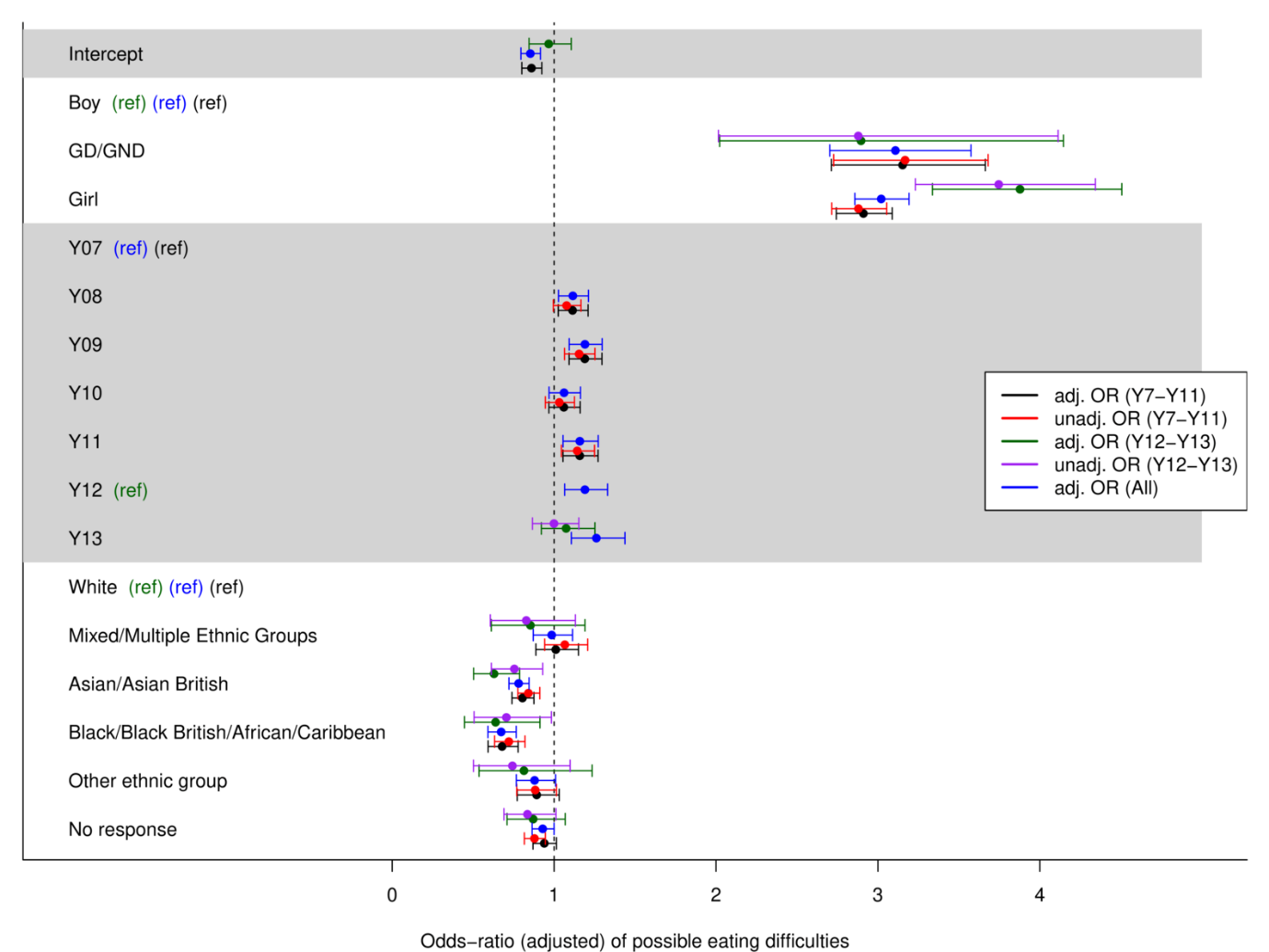
**

**Figure S6. Sensitivity Scenario A equivalent of Figure 4**

**
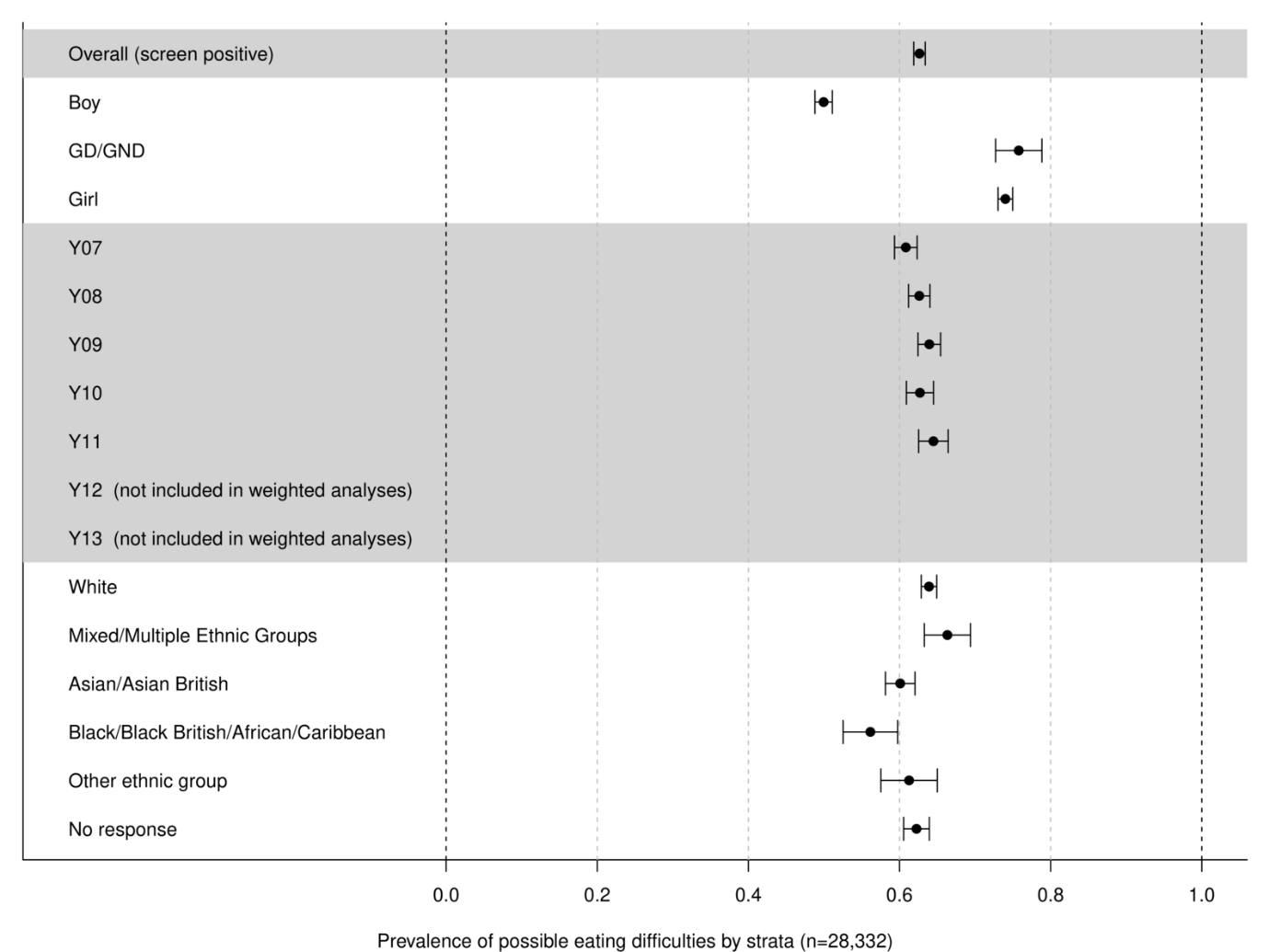
**

**Figure S7. Sensitivity Scenario B equivalent of Figure 4**

**
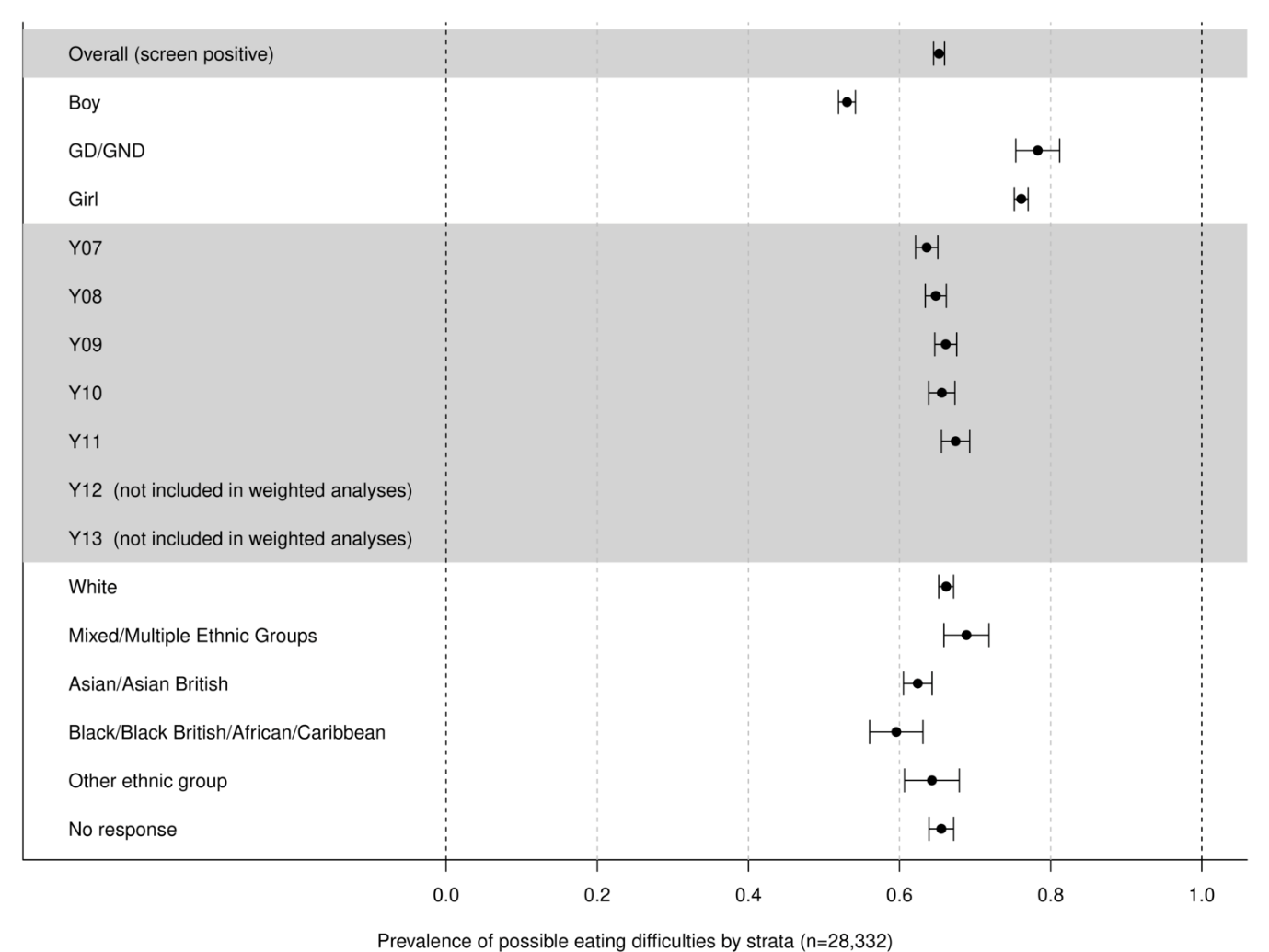
**

**Figure S8. Sensitivity Scenario C equivalent of Figure 4**

**
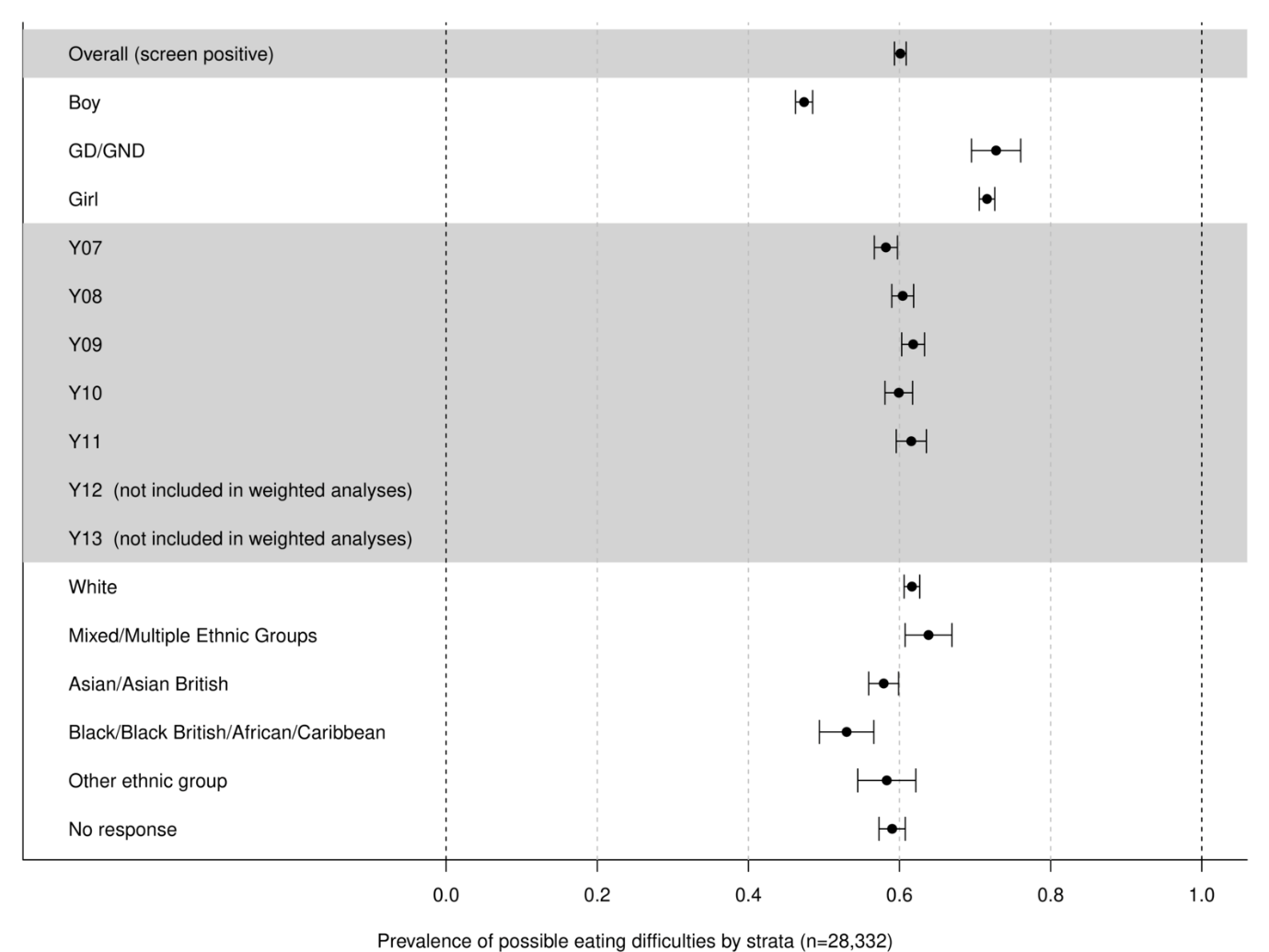
**

**Table S4. Comparison of Sensitivity Scenario Figure 3 estimates**

| **Scenario** | **Covariate** | **SE (adjusted)** | | **SE (unadjusted)** | | **FE (adjusted)** | | **FE (unadjusted)** | | **All (adjusted)** | |
| --- | --- | --- | --- | --- | --- | --- | --- | --- | --- | --- | --- |
|  |  | **Estimate** | **CI** | **Estimate** | **CI** | **Estimate** | **CI** | **Estimate** | **CI** | **Estimate** | **CI** |
| A | No response | 0.977 | (0.903, 1.057) | 0.910 | (0.844, 0.981) | 0.892 | (0.727, 1.095) | 0.854 | (0.705, 1.035) | 0.965 | (0.896, 1.039) |
|  | Other ethnic group | 0.912 | (0.788, 1.054) | 0.901 | (0.784, 1.035) | 0.826 | (0.537, 1.272) | 0.753 | (0.502, 1.129) | 0.898 | (0.781, 1.032) |
|  | Black/Black British/African/Caribbean | 0.691 | (0.602, 0.795) | 0.734 | (0.643, 0.838) | 0.668 | (0.460, 0.970) | 0.733 | (0.519, 1.038) | 0.688 | (0.602, 0.785) |
|  | Asian/Asian British | 0.793 | (0.728, 0.863) | 0.830 | (0.766, 0.901) | 0.617 | (0.491, 0.776) | 0.743 | (0.602, 0.919) | 0.769 | (0.710, 0.834) |
|  | Mixed/Multiple Ethnic Groups | 1.024 | (0.899, 1.166) | 1.082 | (0.955, 1.226) | 0.864 | (0.618, 1.207) | 0.836 | (0.610, 1.146) | 0.998 | (0.883, 1.128) |
|  | White |  |  |  |  |  |  |  |  |  |  |
|  | Y13 |  |  |  |  | 1.074 | (0.918, 1.257) | 1.001 | (0.865, 1.159) | 1.308 | (1.147, 1.492) |
|  | Y12 |  |  |  |  |  |  |  |  | 1.234 | (1.102, 1.382) |
|  | Y11 | 1.180 | (1.075, 1.296) | 1.162 | (1.062, 1.271) |  |  |  |  | 1.181 | (1.075, 1.297) |
|  | Y10 | 1.078 | (0.983, 1.181) | 1.046 | (0.958, 1.142) |  |  |  |  | 1.079 | (0.984, 1.183) |
|  | Y09 | 1.177 | (1.081, 1.282) | 1.141 | (1.052, 1.238) |  |  |  |  | 1.178 | (1.081, 1.283) |
|  | Y08 | 1.101 | (1.013, 1.196) | 1.064 | (0.983, 1.153) |  |  |  |  | 1.103 | (1.014, 1.198) |
|  | Y07 |  |  |  |  |  |  |  |  |  |  |
|  | Girl | 2.971 | (2.800, 3.152) | 2.934 | (2.766, 3.112) | 3.949 | (3.390, 4.602) | 3.807 | (3.276, 4.424) | 3.080 | (2.915, 3.254) |
|  | GD/GND | 3.350 | (2.870, 3.911) | 3.366 | (2.884, 3.928) | 3.035 | (2.097, 4.393) | 3.014 | (2.087, 4.353) | 3.297 | (2.854, 3.808) |
|  | Boy |  |  |  |  |  |  |  |  |  |  |
|  | Intercept | 0.943 | (0.877, 1.014) |  |  | 1.109 | (0.969, 1.270) |  |  | 0.937 | (0.873, 1.006) |
|  |  |  |  |  |  |  |  |  |  |  |  |
| B | No response | 1.019 | (0.943, 1.102) | 0.947 | (0.879, 1.021) | 0.914 | (0.744, 1.121) | 0.874 | (0.720, 1.060) | 1.005 | (0.934, 1.081) |
|  | Other ethnic group | 0.936 | (0.809, 1.084) | 0.922 | (0.801, 1.062) | 0.845 | (0.548, 1.303) | 0.770 | (0.511, 1.159) | 0.922 | (0.802, 1.061) |
|  | Black/Black British/African/Caribbean | 0.714 | (0.622, 0.820) | 0.756 | (0.663, 0.862) | 0.718 | (0.494, 1.044) | 0.780 | (0.549, 1.107) | 0.714 | (0.627, 0.813) |
|  | Asian/Asian British | 0.788 | (0.724, 0.857) | 0.824 | (0.760, 0.893) | 0.613 | (0.488, 0.770) | 0.734 | (0.593, 0.908) | 0.764 | (0.706, 0.828) |
|  | Mixed/Multiple Ethnic Groups | 1.042 | (0.913, 1.189) | 1.101 | (0.970, 1.250) | 0.872 | (0.620, 1.227) | 0.843 | (0.610, 1.164) | 1.015 | (0.896, 1.149) |
|  | White |  |  |  |  |  |  |  |  |  |  |
|  | Y13 |  |  |  |  | 1.074 | (0.920, 1.255) | 1.005 | (0.869, 1.162) | 1.353 | (1.185, 1.544) |
|  | Y12 |  |  |  |  |  |  |  |  | 1.274 | (1.138, 1.426) |
|  | Y11 | 1.199 | (1.092, 1.317) | 1.179 | (1.077, 1.290) |  |  |  |  | 1.199 | (1.092, 1.317) |
|  | Y10 | 1.094 | (0.997, 1.201) | 1.061 | (0.971, 1.159) |  |  |  |  | 1.095 | (0.998, 1.202) |
|  | Y09 | 1.159 | (1.062, 1.264) | 1.124 | (1.034, 1.221) |  |  |  |  | 1.159 | (1.063, 1.264) |
|  | Y08 | 1.080 | (0.994, 1.175) | 1.046 | (0.966, 1.134) |  |  |  |  | 1.082 | (0.995, 1.176) |
|  | Y07 |  |  |  |  |  |  |  |  |  |  |
|  | Girl | 2.938 | (2.768, 3.118) | 2.897 | (2.731, 3.073) | 3.842 | (3.288, 4.490) | 3.702 | (3.175, 4.316) | 3.037 | (2.873, 3.210) |
|  | GD/GND | 3.439 | (2.937, 4.027) | 3.454 | (2.951, 4.043) | 3.011 | (2.076, 4.368) | 2.989 | (2.065, 4.325) | 3.368 | (2.910, 3.899) |
|  | Boy |  |  |  |  |  |  |  |  |  |  |
|  | Intercept | 1.057 | (0.982, 1.136) |  |  | 1.302 | (1.138, 1.488) |  |  | 1.052 | (0.979, 1.130) |
|  |  |  |  |  |  |  |  |  |  |  |  |
| C | No response | 0.939 | (0.870, 1.015) | 0.879 | (0.816, 0.946) | 0.871 | (0.709, 1.069) | 0.835 | (0.690, 1.012) | 0.930 | (0.864, 1.000) |
|  | Other ethnic group | 0.892 | (0.772, 1.031) | 0.883 | (0.769, 1.015) | 0.814 | (0.536, 1.235) | 0.743 | (0.502, 1.099) | 0.880 | (0.767, 1.009) |
|  | Black/Black British/African/Caribbean | 0.679 | (0.592, 0.778) | 0.720 | (0.633, 0.820) | 0.638 | (0.447, 0.913) | 0.705 | (0.506, 0.983) | 0.673 | (0.591, 0.766) |
|  | Asian/Asian British | 0.805 | (0.740, 0.875) | 0.841 | (0.776, 0.912) | 0.629 | (0.503, 0.787) | 0.755 | (0.613, 0.930) | 0.781 | (0.721, 0.845) |
|  | Mixed/Multiple Ethnic Groups | 1.010 | (0.887, 1.150) | 1.066 | (0.942, 1.207) | 0.854 | (0.612, 1.191) | 0.828 | (0.606, 1.132) | 0.985 | (0.871, 1.114) |
|  | White |  |  |  |  |  |  |  |  |  |  |
|  | Y13 |  |  |  |  | 1.074 | (0.921, 1.252) | 0.999 | (0.866, 1.153) | 1.261 | (1.106, 1.438) |
|  | Y12 |  |  |  |  |  |  |  |  | 1.190 | (1.065, 1.330) |
|  | Y11 | 1.158 | (1.055, 1.272) | 1.143 | (1.045, 1.250) |  |  |  |  | 1.159 | (1.055, 1.273) |
|  | Y10 | 1.060 | (0.968, 1.161) | 1.032 | (0.946, 1.126) |  |  |  |  | 1.061 | (0.969, 1.163) |
|  | Y09 | 1.190 | (1.093, 1.295) | 1.154 | (1.064, 1.252) |  |  |  |  | 1.191 | (1.093, 1.297) |
|  | Y08 | 1.114 | (1.026, 1.210) | 1.077 | (0.995, 1.166) |  |  |  |  | 1.116 | (1.027, 1.212) |
|  | Y07 |  |  |  |  |  |  |  |  |  |  |
|  | Girl | 2.910 | (2.743, 3.088) | 2.880 | (2.715, 3.054) | 3.878 | (3.337, 4.506) | 3.746 | (3.231, 4.343) | 3.020 | (2.858, 3.192) |
|  | GD/GND | 3.152 | (2.713, 3.664) | 3.168 | (2.727, 3.680) | 2.896 | (2.022, 4.146) | 2.879 | (2.015, 4.113) | 3.108 | (2.703, 3.575) |
|  | Boy |  |  |  |  |  |  |  |  |  |  |
|  | Intercept | 0.860 | (0.801, 0.924) |  |  | 0.967 | (0.845, 1.106) |  |  | 0.853 | (0.795, 0.916) |

**Table S5. Comparison of Sensitivity Scenario Figure 4 estimates**

| **Scenario** | **Covariate** | **Estimate** | **CI** |
| --- | --- | --- | --- |
| A (0.0) | No response | 0.6224 | (0.6054, 0.6395) |
|  | Other ethnic group | 0.6127 | (0.5753, 0.6501) |
|  | Black/Black British/African/Caribbean | 0.5614 | (0.5253, 0.5975) |
|  | Asian/Asian British | 0.6009 | (0.5814, 0.6205) |
|  | Mixed/Multiple Ethnic Groups | 0.6633 | (0.6328, 0.6938) |
|  | White | 0.6390 | (0.6288, 0.6492) |
|  | Y11 | 0.6449 | (0.6253, 0.6644) |
|  | Y10 | 0.6271 | (0.6091, 0.6450) |
|  | Y09 | 0.6394 | (0.6245, 0.6544) |
|  | Y08 | 0.6261 | (0.6119, 0.6403) |
|  | Y07 | 0.6084 | (0.5934, 0.6234) |
|  | Girl | 0.7401 | (0.7305, 0.7498) |
|  | GD/GND | 0.7578 | (0.7271, 0.7885) |
|  | Boy | 0.4996 | (0.4881, 0.5110) |
|  | Overall (screen positive) | 0.6264 | (0.6188, 0.6340) |
| B (+0.2) | No response | 0.6554 | (0.6391, 0.6717) |
|  | Other ethnic group | 0.6429 | (0.6067, 0.6791) |
|  | Black/Black British/African/Caribbean | 0.5958 | (0.5605, 0.6311) |
|  | Asian/Asian British | 0.6242 | (0.6052, 0.6431) |
|  | Mixed/Multiple Ethnic Groups | 0.6886 | (0.6588, 0.7184) |
|  | White | 0.6617 | (0.6519, 0.6716) |
|  | Y11 | 0.6742 | (0.6555, 0.6929) |
|  | Y10 | 0.6560 | (0.6385, 0.6735) |
|  | Y09 | 0.6612 | (0.6467, 0.6757) |
|  | Y08 | 0.6480 | (0.6342, 0.6618) |
|  | Y07 | 0.6359 | (0.6212, 0.6507) |
|  | Girl | 0.7611 | (0.7519, 0.7703) |
|  | GD/GND | 0.7830 | (0.7540, 0.8120) |
|  | Boy | 0.5305 | (0.5193, 0.5417) |
|  | Overall (screen positive) | 0.6523 | (0.6450, 0.6595) |
| C (-0.2) | No response | 0.5902 | (0.5728, 0.6077) |
|  | Other ethnic group | 0.5831 | (0.5447, 0.6215) |
|  | Black/Black British/African/Caribbean | 0.5300 | (0.4940, 0.5660) |
|  | Asian/Asian British | 0.5791 | (0.5593, 0.5988) |
|  | Mixed/Multiple Ethnic Groups | 0.6384 | (0.6075, 0.6693) |
|  | White | 0.6164 | (0.6061, 0.6267) |
|  | Y11 | 0.6156 | (0.5956, 0.6355) |
|  | Y10 | 0.5991 | (0.5807, 0.6174) |
|  | Y09 | 0.6181 | (0.6029, 0.6332) |
|  | Y08 | 0.6042 | (0.5897, 0.6187) |
|  | Y07 | 0.5820 | (0.5667, 0.5973) |
|  | Girl | 0.7158 | (0.7056, 0.7260) |
|  | GD/GND | 0.7278 | (0.6952, 0.7604) |
|  | Boy | 0.4737 | (0.4624, 0.4850) |
|  | Overall (screen positive) | 0.6011 | (0.5933, 0.6089) |
